# An Emergency Response System: Construction, Validation, and Experiments for Disaster Management in a Vehicular Environment

**DOI:** 10.3390/s19051150

**Published:** 2019-03-07

**Authors:** Kishwer Abdul Khaliq, Omer Chughtai, Abdullah Shahwani, Amir Qayyum, Jürgen Pannek

**Affiliations:** 1 Department of Production Engineering, University of Bremen, 28539 Bremen, Germany; 2 BIBA-Bremer Institut für Produktion und Logistik GmbH, Hochschulring 20, 28359 Bremen, Germany; pan@biba.uni-bremen.de; 3 Department of Electrical and Computer Engineering, COMSATS University Islamabad, Wah Campus, Islamabad 45550, Pakistan; umer@ciitwah.edu.pk; 4 Department of Physics and Electrical Engineering, University of Bremen, 28359 Bremen, Germany; shahwani@uni-bremen.de; 5 Faculty of Engineering, Capital University of Science and Technology Islamabad, Islamabad 44000, Pakistan ; aqayyum@ieee.org; 6 Department of Production Engineering, University of Bremen, 28359 Bremen, Germany

**Keywords:** IEEE 802.11p, VANET, Ad hoc networks, Raspberry Pi, safety applications, disaster management, emergency response, TCP/IP architecture, message dissemination, information exchange

## Abstract

Natural disasters and catastrophes not only cost the loss of human lives, but adversely affect the progress toward sustainable development of the country. As soon as disaster strikes, the first and foremost challenge for the concerned authorities is to make an expeditious response. Consequently, they need to be highly-organized, properly-trained, and sufficiently-equipped to effectively respond and limit the destructive effects of a disaster. In such circumstances, communication plays a vital role, whereby the consequences of tasks assigned to the workers for rescue and relief services may be streamlined by relaying necessary information among themselves. Moreover, most of the infrastructure is either severely damaged or completely destroyed in post-disaster scenarios; therefore, a Vehicular Ad Hoc Network (VANET) is used to carry out the rescue operation, as it does not require any pre-existing infrastructure. In this context, the current work proposes and validates an effective way to relay the crucial information through the development of an application and the deployment of an experimental TestBed in a vehicular environment. The TestBed may able to provide a way to design and validate the algorithms. It provides a number of vehicles with onboard units embedded with a credit-card-size microcomputer called Raspberry Pi and a Global Positioning System (GPS) module. Additionally, it dispatches one of the pre-defined codes of emergency messages based on the level of urgency through multiple hops to a central control room. Depending on the message code received from a client, the server takes appropriate action. Furthermore, the solution also provides a graphical interface that is easy to interpret and to understand at the control room to visualize the rescue operation on the fly.

## 1. Introduction

Forest fires, floods, storms, volcanic eruptions, and droughts left about 606 billion people dead and nearly 4.1 billion injured or homeless around the globe between 1995 and 2015, as specified in a report published by the United Nations Office for Disaster Risk Reduction (UNISDR) [1,2]. It accentuates that such disasters have caused economic damage worth over 2.0 trillion dollars, and that it leaves mankind vulnerable and helpless.

When a disaster occurs, most of the existing infrastructure is either completely destroyed or severely damaged. Thus, making any use of pre-deployed communication services is almost impossible. More often than not, such disasters strike areas which are far from metropolitans, where the facilities are already at a minimal level. In such places, the effectiveness of any underway rescue operation is quite limited. Hence, researchers are constantly seeking methods whereby accurate and timely information could flow among the rescue teams, which can boost work-efficiency and lead to favorable results. The deployment of a cost-effective and reliable communication setup in such areas can make a huge difference. Fortunately, we are witnessing a revival of Vehicular Ad Hoc Networks (VANETs) as a concept that provides a simple and commercially viable solution to the predicament of emergency responses in post-disaster management scenarios [3,4,5]. VANET is superior to other potential technologies because of its readiness, low complexity, and comparably better coverage. It supports road safety, and traffic efficiency and management, as well as infotainment applications [6,7]. In the very near future, the emergence of non-safety applications may generate a big market revenue by virtue of an increase in the number of vehicles that are equipped with sophisticated onboard wireless devices [6,8,9]. Nevertheless, the main applications of VANET, even today, revolve around safety and security.

Although all the above-mentioned applications are significant in their own ways, we must keep in mind that the most critical role of VANETs lies in emergency response and disaster management systems [7,10,11] (these are also the key points of this study). Over the years, natural disasters have exponentially been increased, partly because of the change in global weather conditions and fast-growing industrialization of developing countries. This has put the focus back to the best use of today’s highly-advanced technologies and the availability of solutions to long-standing scientific queries. In our previous study [11], the design of a prototype for a centralized emergency response system was presented, which works fine with the help of a Global Positioning System (GPS) under fixed circumstances using VANET. The challenging part of the current study is to design and develop an application for disaster management using an emergency response system with a user-friendly interface, and to implement the TestBed with the ad hoc configuration. This study would also be useful to the scientific society due to the goals and objectives of the designed applications. Since the proposed study was applied in a vehicular environment, it can be used to represent real-time statistics of an emergency response system, along with the disaster statistics of an affected area. It may prove effective for the scientific society as it shows the round-trip-time with reference to the number of hops along the route from the source to destination. The society may also take advantages to know about the reliability factor in terms of packet delivery ratio based on the hop-count value along the route within the network. Moreover, it might be helpful for the scholars and the scientific society to relate the delay for the emergency messages based on the characteristics of the network. Furthermore, this work proposes a TestBed and provides the flexibility to configure various mobility scenarios in a vehicular environment. Moreover, it carries out the evaluation of the proposed application on the configured TestBed. Furthermore, the proposed prototype application is examined through experimental validation.

The remaining paper is organized as follows: Section 2 briefly looks at the current literature regarding the applications of VANET in post-disaster management scenarios, and describes some solutions on how we can further improve them, especially in terms of the practicality of such propositions. Section 3 presents the implementation of the proposed idea in detail. Section 4 explains the ad hoc configuration of the TestBed and examines the functionality of the designed application, along with the validation of the proposed TestBed. Finally, Section 5 concludes the study, and also discusses future work.

## 2. Literature Review

The first 72 h after the occurrence of a disaster are the most critical hours, as chances of finding survivors after this time start to swiftly decrease. The authors in [12] thus suggest that communication among the first few responders may play a pivotal role in achieving efficient coordination and may help avoid any additional deaths. Moreover, the authors followed a systematic approach to make readers understand the possibilities offered by various multi-hop ad hoc paradigms and technologies. The paper also discussed the analysis and simulation of different mobility models and suggested that the use of smartphones in post-disaster scenarios is one of the best possible options due to its common use. But although it gives the reader a very good understanding of the current study in a defined scope, it fails to come up with any practical real-world solution. An on-board vehicle-to-vehicle (V2V) multi-hop wireless networking system as a TestBed was developed in [13] to evaluate the real-world performance of telematics applications. The TestBed employs a differential GPS receiver, an IEEE802.11a radio card modified to emulate the Direct Short-Range Communication (DSRC) standard, a 1xRTT cellular-data connection, an onboard computer, and audio-visual-based equipment. The main aim was to evaluate the feasibility of high-speed inter-vehicular communication and to develop routing protocols for highly mobile networks. Vehicles communicate with each other via a magnetic-mounted wireless antenna with line-of-sight (LOS) reception. In essence, this application is suitable for V2V communication in an urban scenario, where vehicles move at a normal speed. However, in our case, mobility is not very dynamic, as vehicles are confined within an immediate disaster area. Moreover, unlike this TestBed, our work does not rely on any Internet support.

The authors in [14] presented DistressNet, a system that addresses the need for search-and-rescue workers after a natural disaster, based on an urban setting. The system integrates inexpensive and heterogeneous battery-powered Commercial Off-The-Shelf (COTS) devices, like smart phones and low-power sensors, into easy-to-deploy architecture. The overall efficiency of the system could be improved by placing sensors at certain optimal locations in the deployment area. Although the authors performed a separate evaluation for different parts of the system, no comprehensive TestBed implementation was carried out. Moreover, the dominant stack used in DistressNet as a networking backbone is IEEE 802.11a/b/g/n with an Independent Basic Service Set (IBSS) mode. Explicitly Parallel Instruction Computing (EPIC) motes were selected as hardware platforms, but IEEE 802.11 support is not widely available for such devices, thus severely restricting their practicality.

The authors in [15] proposed a Radio-Frequency Identification (RFID)-based Indoor Localization System (ILS) for the first few responders in emergency scenarios. The authors performed various tests under different scenarios to assess the capabilities of their proposed system. However, the system turns out to have a high dependency on metrics like the number of RFID tags, the minimum read distance of RFID tags, installation angles of tags, and the RFID reader positioning on the rescuer. Therefore, the proposed system is less effective in unpredictable environments. The authors in [16] suggested a server-less peer-to-peer communication network (P2Pnet), which is based on a Mobile Ad hoc Network (MANET) to support temporary group communication and an information-sharing network. According to the authors, if some of the nodes in the network are acting as gateways required to access the Internet service through some satellite communication capability, the service is passed to all the other nodes in the network. Additionally, the paper suggested the use of Walkie-Talkie hand-held devices, or WiFi-ready mini-notebook PCs as communicating nodes, which shall rely on portable generators to supply power. Although it might work in cases where the severity of the disaster is not high, it will probably not be a feasible solution in most disaster scenarios. The authors also proposed a Rescue Information System for Earthquake Disasters (RISED), which aids in disaster assessment through seismic-related information. The objective of RISED is to provide up-to-date and accurate rescue-related information, such as disaster locations, possible damage to lives and construction, available rescue-and-relief resources, and the shortest way to disaster spots, etc. This system, however, was not practically deployed.

For local communication and information collection, authors in [17] proposed and deployed an Integrated Emergency Communication System (IECS) for disaster-struck areas using a Wireless Sensor Network (WSN) and MANET. However, for remote communication, the satellite gateway is used in disaster-safe areas. Furthermore, they proposed an Integrated Emergency Service System (IESS) to provide a variety of emergency management services to people involved in disaster relief missions. MANET is formed using an IEEE 802.11s wireless mesh network, which is comprised of Mesh Access Points (MAPs) and Mesh Clients (MCs). Based on the network conditions and service requirements (local or remote), the mobile end-user can select a communication path either through MANET or through the cellular network (if available), or by using a satellite network. Although it appears feasible in theory, however, neither any practical work nor any actual implementation has been carried out in this regard. In [18], the authors proposed a post-disaster emergency response system using a MANET TestBed. They shared some files from one laptop to another and demonstrated their proposed system. In spite of the fact that the implementation proposed in [18] confirms the feasibility of systems developed for real-world scenarios, it does not accomplish much in terms of a concrete prototype configuration and implementation. Since there is no exchange of relevant information—for instance, they either used geographical coordinates of the disaster area or consolidated the request of any relief-related resource, thus meaning that the scope of this TestBed is extremely limited.

In [19], the authors proposed a reconfigurable TestBed development using a Field-Programmable Gate Array (FPGA). They simulated behavioral models for different VANET classifications—for example, the Xilinx ISE (Integrated Synthesis Environment) Suite was used as a simulation tool to extract results by generating and implementing a Verilog code. According to the authors, using dedicated software applications like SUMO (Simulation of Urban MObility) or GrooveSim with VANET are inefficient in terms of time, as these applications have their own limitations. Moreover, they suggest that in their proposed FPGA-based approach, the same chip can be reprogrammed very easily for upgradation purposes if the user wants any additional feature in a customized vehicular network. The motivation to implement an FPGA-based TestBed rather than a regular microcontroller-based TestBed was to achieve higher performance due to FPGAs’ parallel-processing mechanism, optimized power requirements, cost reduction, remote access, and runtime hardware reconfigurability for real-time applications. Like the ideas described earlier, this system is also limited to simulations only. Any real-world TestBed implementation of the aforementioned system is yet to be carried out. In [20], the authors discussed an open TestBed that integrates an ad hoc V2V communication and a wireless mesh back-haul, which is based on MobiMESH (Wireless Mesh Network and Mobility Project) hardware/software solutions.

The above-mentioned research is based on theoretical analysis, design, and/or simulations being carried out to interrogate various applications and multi-hop ad hoc routing of VANETs in post-disaster emergency response situations. However, these ideas have no practical implementations to assist in real-world cases of post-disaster rescue-and-response efforts. Therefore, in this paper, we intend to provide a test-bed application along with the configuration and implementation of VANET, through dynamic multi-hop ad hoc routing without any dependency on the Internet backbone. This is achieved through a suitable combination of software and hardware components that work together to form a comprehensive system for emergency response in disaster management operations. The proposed application has the ability to support the communication need of the first 72 h to reduce post-disaster effects and help disaster relief operations. The design is simple and user-friendly. Further details on application design, selected hardware, and implementation are given in Section 3.

## 3. System Design and Implementation

Within the scope of current study and its implementation as a TestBed, we comprehensively deploy the developed prototype application and the ad hoc network implementation, along with its configuration in order to understand the actual outcomes and certain constraints offered by VANET. This section discusses the proposed arrangement and the hardware used for successful TestBed implementation.

### 3.1. Preamble

An ad hoc network with flat configuration typically bypasses the need of an external router or an access point and provides a direct path to the endpoints in a network. When we consider emergency situations in far-flung areas, traditional networks become very expensive and are difficult to deploy. In contrast to this, medical or rescue teams may utilize IEEE802.11 radio adapters that are available in their laptops or Personal Digital Assistants (PDAs) as soon as they arrive at the scene.

An ad hoc network may be utilized in so many ways—for example, if there is a fire in a big forest, it would be much easier for the team of fire-fighters to deploy an ad hoc network, instead of building an entire infrastructure in such an area. To a certain limited extent, it has been established that nodes in an ad hoc network are usually not powerful enough and contingent on the transmission range [21]. However, when we combine these transmission ranges together, we can form a sustainable network based on a larger geographical area. The communication between nodes within the network can then be achieved through a routing protocol. Consequently, the nodes would create a transmission-cloud to exchange information among themselves, through some sort of structured routing algorithm.

### 3.2. Proposed System

The proposed prototype mainly includes two entities—a server and a client. The client part is integrated on the On-Board Unit (OBU) that is basically deployed in a vehicle or in a hand-held device which is used by a volunteer during rescue operations. The server part is the control room which is primarily a central entity to visualize all the statistics that are being collected in the affected area. It has a user-friendly interface for data visualization and a concurrency-based Python code that generates response against specific requests initiated by the rescue team members from their hand-held devices, hence achieving bi-directional communication based on the Transmission Control Protocol/Internet Protocol (TCP/IP) suite of the Open Systems Interconnection (OSI) model. The received information comprises a structured data format, which includes the coordinates of the exact location acquired from a GPS sensor attached with the transmitting node. All of this will be explained in detail in the sections to follow.

**Figure 1:**
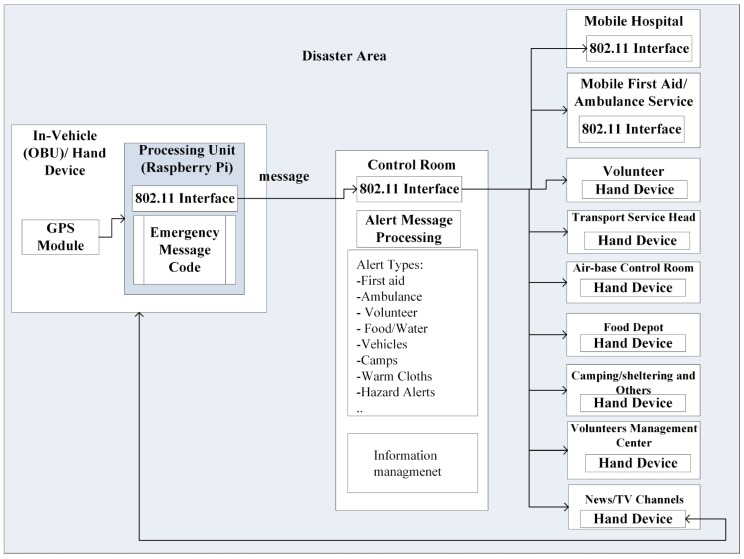
Emergency Response Plan in Disaster Scenario.

The proposed emergency response plan is illustrated in Figure 1. A volunteer who is moving around the affected area generates a peremptory request in a message for certain need at the incident location of a seriously injured patient. The OBU transmits a message by encapsulating the GPS coordinates of the location of interest through several hops to the destination, i.e., the Control Room. Here, the Raspberry Pi module, in addition to the GPS sensor, is used as a processing unit. The Control Room receives the message through a wireless interface, and processes the request according to its severity. Just as in a real-world scenario, as some messages can be more critical for life-saving measures than others, they shall be treated accordingly. Control room then performs the desired action by either sending the message to another entity such as a mobile hospital, or provides a notification on the user interface with messages that are less urgent. Since small-size packets like safety/alert messages move quickly and reliably in the network as compared to large-size packets, such as multimedia, safety/alert messages are mapped into codes. The alert messages, their types, and respective codes are shown in Table 1.

**Figure 2:**
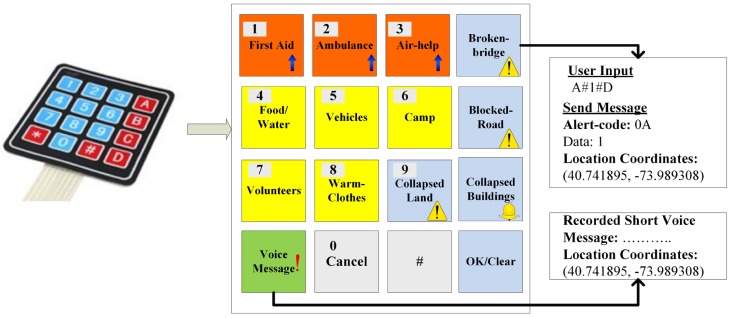
An example of inserting data at the Client interface.

Now, instead of sending the whole text message, only the message code is sent, as shown in Table 1, which is routed within the network. A typical example of how a message is formatted and sent is shown in Figure 2 using a keypad.

In order to use the application, a user is required to press a button according to the need—for example, a button with code **4** followed by a **#** sign, which is used as a separator, indicates the requirement of water or food at a certain location. Another button can then be pressed if a fixed quantity is desired—for example, a button with code **20** indicates food packages or water bottles. More than one message can also be concatenated in one go by pressing more buttons each time with a **#** separator between them. When the entire message is compiled, **D** is pressed to indicate the end of a message. This entire formatted text is then wrapped in a JavaScript Object Notation (JSON) wrapper and sent to the destination.

Since the central server at the Control Room is constantly coordinating and interacting with various departments involved in rescue operations to provide post-disaster management services, its actions can be mapped according to the user requests it receives. This can be seen in Table 2.

The front-end Graphical User Interface (GUI) at the Control Room shows the map of the disaster area, the list of departments collaborating in the rescue activities, the log of all the operations underway, and the status of resources at disposal. This data visualization makes it easier for the authorities to make effective and timely decisions based on accurate and up-to-date information. The GUI of the server-side application is developed in Python using the Tkinter module in Python.

### 3.3. Implementation

At Bremer Institut für Produktion und Logistik GmbH, we deployed the implemented ad hoc network. The deployed setup forms a network that comprises of Raspberry Pi (a low-cost micro-computer with an open-source operating system) nodes, which can either transmit their own packets or forward packets generated by other Raspberry Pies, hence acting as Relay Nodes (RNs). GoPiGo (General Purpose Input Output) robot cars fabricated by Dexter Industries, which comes with their motherboards and can be assembled manually, are used to provide motion to the Raspberry Pi nodes. Each GoPiGo robot car has a D-Link Nano USB Adapter Dongle that provides support for routing or forwarding the received contents. The node acting as an OBU, additionally has a GPS sensor and a keypad for user input.

The hardware and software had to be chosen carefully to meet the necessary requirements of the task. Therefore, the following hardware was used for successful TestBed implementation:

#### 3.3.1. Go-Pi-Go Robots

**Figure 3:**
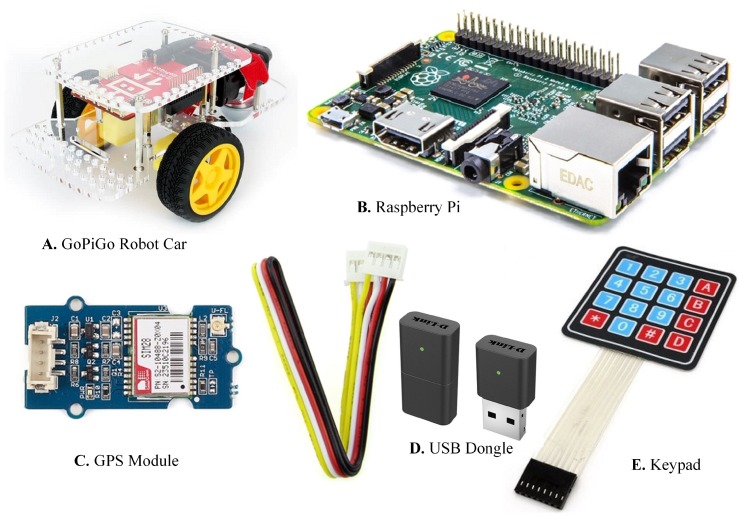
Hardware used in designing an emergency response system.

To encompass mobility in Raspberry Pi-based nodes, GoPiGo robots by Dexter Industries were used, as shown in Figure 3A.

#### 3.3.2. Raspberry Pi 2

The choice of hardware technology plays a significant role because of its usefulness and commercial availability; however, it is also constrained by the cost. For the specified reasons, we chose Raspberry Pi 2 model B, a credit card-sized microcomputer with basic hardware processing, as shown in Figure 3B. It is relatively cheap, consumes less power compared to other microcomputers, and is widely available [22,23,24]. Raspberry Pi acts as a processing unit that acquires user data through a keypad and the data about the location from a GPS sensor. It then passes the acquired data to the client application, which is running on a Linux Debian operating system. It is equipped with a powerful application processor [25,26,27]. The client-end sends 4 × 4 keypad’s user-data and the GPS sensor’s location data serially to the General Purpose Input/Output (GPIO) pins of the Raspberry Pi. This data then gets manipulated through a client application, whereas the server receives this data using web sockets.

#### 3.3.3. Grove GPS Sensor

For accurate and timely emergency response services, it is assumed that the exact location of the victims or a collapsed building or similar medical emergencies is known. We used the Grove GPS Breakout board, as shown in Figure 3C, to identify the desired location. It is a cost-efficient and field-programmable gadget, and uses satellite signals to determine the current position, time, and velocity of the OBU. To test the system in an indoor environment, the external antenna needs to be attached with the GPS receiver—otherwise, the GPS receiver may receive deteriorated signals.

#### 3.3.4. USB Adapter Dongle

Several adapters have been tested with Raspberry Pi, but the ad hoc support was not available. Therefore, we selected the DWA-131 (D-link Wireless N Nano USB Adapter) as shown in Figure 3D, which is a convenient wireless connectivity solution for microcomputers, PDAs (Personal Digital Assistants), and desktop or notebook PCs. It comes with the stable driver for Raspberry Pi, which needs to be installed prior to use.

#### 3.3.5. 4 × 4 Matrix Keypad

A small 4 × 4 matrix keypad, as indicated in Figure 3E, provides the most basic user-input capability which serves the main messaging task for the TestBed implementation.

### 3.4. Client Server Model

In a dynamic ad hoc network that we have used in our proposed TestBed, these OBUs in vehicles make service requests to the Control Room, which acts as a central “server”. As the network is wireless and the server is a singular entity, all clients cannot reach it directly—they might have to traverse a long path before their message reaches the server; thus comes the concept of “multi-hopping”, where message might have to pass through multiple nodes to eventually reach the destination.

In such a scenario, the most efficient approach is to handle each aspect of the implementation separately. For this purpose, two parallel applications were developed:

- The server-side application communicates with multiple clients and sends a response to their individual needs, all at once.
- The client-side application communicates with server and sends a request message in which a rescue team member inserts a message code indicates the required service at a specific location.

#### 3.4.1. Python—A High-Level Programming Paradigm

The entire application is built on Python, which is an elegant open-source, cross-platform, high-level, dynamic interpreted language [28]. Being a multi-platform-supported language, it runs equally well on Windows and Linux. Debian, the Raspberry Pi operating system, contains a Python application out-of-the-box. Python is a very powerful, yet flexible and widely-used procedural language that serves various purposes, such as web and desktop application development, system administration tasks, scientific research, data analysis and management, and event-driven applications.

Henceforth, both client-side and server-side applications are briefly explained, along with their key concepts for successful completion of the task.

#### 3.4.2. Client-Side Application

**Figure 4:**
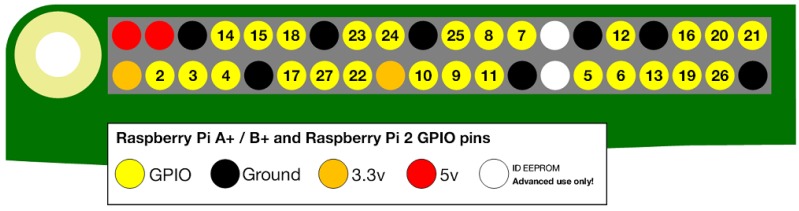
General-purpose input/output pins of Raspberry Pi model B+ [29].

A brief overview of the main Python libraries used in this client-side application is as follows:

- **Socket:** This module provides low-level inter-process communication on Layer 3 (Network layer) of the TCP/IP (Transmission Control Protocol/Internet Protocol) architecture by implementing socket system calls. A socket, in the simplest term, is a gateway that provides point-to-point, two-way communication between two processes running on two separate endpoints.
- **RPi.GPIO:** This is a Python module that controls the general-purpose I/O pins on a Raspberry Pi. The configuration of GPIO pins on a Raspberry Pi can be examined in Figure 4. We can retrieve the values inserted by the user through an attached keypad, by setting up the rows and columns of the 4 × 4 Matrix keypad as the input and output.
- **Threading:** A thread of execution is the smallest sequence of programmed instructions that can be independently managed by a system scheduler, which is typically a part of the Operating System (OS) [30]. The thread waits for the user to press a button, while the GPS monitors the current location. Both of these activities need to be performed simultaneously, and this can be achieved by opening two parallel threads that can work independently.

A separate “GpsController” class works with a GPS daemon to periodically collect the latitude and longitude values from the satellite. These values can be pulled out whenever required by declaring an object of this class and calling a “next()” method on it. To wrap it up, whenever a client-code is executed, the node gets connected to either the nearest intermediate node or directly to the server, provided that the node is in the vicinity. Once a TCP-based connection is up and running, the data transmission can take place. Since the data can have a random size depending upon the input fed by the rescue team member, it can just be an emergency call that requires you to dispatch an ambulance or give some additional information regarding the collapse of a building or a blockage of some nearby road. In order to bring flexibility into the picture, a Java Script Object Notation (JSON) wrapper is used, which can adequately manage a message of variable length. After all the data is nicely compiled and packaged, it is sent through the socket interface.

#### 3.4.3. Server-Side Application

A TCP connection is a reliable one-to-one communication window that facilitates bi-directional talk between two terminals. However, in our case, as previously mentioned in Section 3.4, a server is required to send a response to all the queries sent by the clients at any given time. Since a single socket connection

between a client and the server is exclusive, no other client can share this channel. For the communication to happen between the server and a client, while there is already an active communication channel, we have to rely on a concept called “Concurrency”. Hardware exception handlers, processes, and Unix signal handlers are all familiar examples of Concurrency in a computer system. In this context, a concurrent server is one that creates a separate logic flow for each client. Nowadays, systems are equipped with multi-core processors that contain multiple CPUs to handle various tasks at once. Applications that run in such machines are faster and executed in parallel rather than being interleaved, as used in single-core systems. There can be three different approaches to dealing with concurrency in modern systems. These are:

- **Processes:** The kernel schedules and maintains each logical control flow as a process. If processes need to exchange information with one another to complete a particular task, then they need to perform some kind of inter-process communication due to their individual virtual address space.
- **I/O multiplexing:** In this form of concurrency, a program is a single process where all logical flows share the same address space. The logical flows are modeled as state machines by the main program, which explicitly makes state-to-state transitions according to the data arriving on file descriptors.
- **Threads:** Threads are a hybrid of the two earlier approaches, and scheduled by the kernel as process flows, it shares the same virtual address space, like I/O multiplexing flows [31].

In prototype application implementation, we used “threads” as a means to provide multi-tasking capability. This abstraction is provided by Python 2.7 in the form of a module called “concurrent.futures”. This module provides a high-level interface for asynchronous execution of “callables” [32]. This execution can be carried out either by threads using a “ThreadPoolExecutor” or by processes using a “ProcessPoolExecutor”. We chose the former, as it is generally preferred for I/O-bound applications.

#### 3.4.4. Graphical User Interface

In a post-disaster management scenario, the control room is occupied by strictly professional individuals who have the ability and authority to make critical decisions in a real-time environment that can cause a sizable impact on the overall operating results. In order to do so, a visual image of everything that is taking place in and around the disaster-struck area is needed. Therefore, visualizing the data being received from OBUs mounted on vehicles or hand-held devices in possession of volunteers and rescue staff, in a meaningful way, is one important aspect of this project. For this purpose, we used the simplest and most straight-forward Python module for the graphical representation of data, called Tkinter.

**Figure 5:**
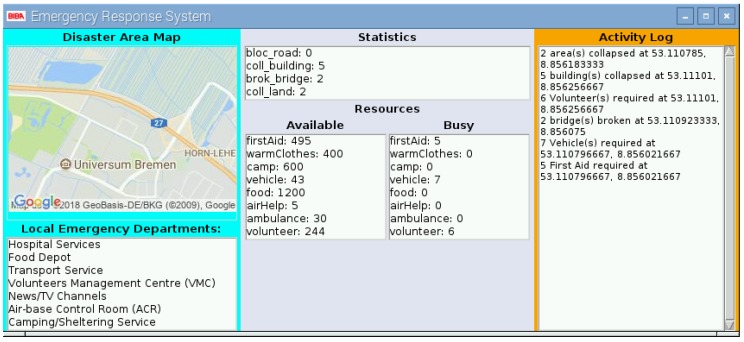
Graphical User Interface (GUI) using Python’s Tkinter library.

GUI is divided into three blocks, as can be seen in Figure 5, each with a very specific responsibility.

- The upper part of the Left Block is responsible for showing the map of the disaster area, while the lower part has the list of all the relevant departments that takes part in the rescue-and-response efforts;
- The top part of the Central Block presents the statistics of the damage caused by the calamity, such as the collapsed area, blocked roads, or broken bridges, while the bottom part shows the current availability of the resources at disposal;
- The Right Block is the activity log where all the alert messages are displayed.

The map present on the GUI was captured from an open-source Application Programming Interface API provided by Google [33].

## 4. Ad Hoc Routing: Configuration, Scenarios, and Results

In essence, an ad hoc network has a flat typological structure with dynamic interconnection of nodes, where each node can communicate with as many nodes as possible in the network through the intermediate or relay nodes, to efficiently forward the information to the desired destination. The flow of information in the network from one endpoint to another can be achieved either by the use of flooding or routing techniques. By implementing routing techniques, a message can hop multiple nodes to ultimately reach its destination. To ensure that there is always a path between the source and destination, the network must reconfigure itself periodically through the use of some routing techniques in order to counter any broken links. An ad hoc network comes with its own set of challenges—since the nodes tend to be mobile, routing algorithms are required to answer issues raised by the mobility of nodes. Luckily in the implemented TestBed, the nodes are OBUs, mounted in vehicles. Since vehicles or nodes are not random in their movement, they, in fact, follow a predefined path, such as a road. Hence, routing can be managed relatively easily.

To test the designed application and the implemented TestBed, it is necessary to consider a routing protocol to configure the TestBed. For this purpose, a routing protocol Better Approach To Mobile Ad hoc Networking (BATMAN). It is a multi-hop mobile ad hoc routing protocol, and was developed by the German Freifunk community. It is a non-commercial open grassroots initiative to support free computer networks in the German region, through which a term known as Collective Intelligence emerged in early 2007. The German Freifunk community has also started to tinker with the idea of routing data on Ethernet Layer (Layer 2) of the TCP/IP Model, instead of Network Layer (Layer 3). This approach provides a virtual interface where packets get transported on their own without maintaining or manipulating any routing tables. This concept increases transparency and takes the burden of routing table management off the shelf. To differentiate this new approach from BATMAN protocol which are already available, they put an ADV at the end, to denote that it is “advanced” [34].

### 4.1. Configuring BATMAN-ADV Protocol

Since the nodes in the TestBed are based on Raspberry Pi, which operate on a Linux Debian distribution (Raspbian Stretch, in this case), the starting point had to be the configuration of the operating system on Pies through raspi-config, and basic ad hoc routing in Linux. Once the basic installation and configuration of Raspberry Pi OS and the ad hoc network [35] was done, respectively, BATMAN-ADV protocol was configured on all Pies. The hands-on settings for configuring BATMAN-ADV is available at the project Git repository in [36].

### 4.2. Scenarios and Results

Once everything is completed, the last step of a successful TestBed implementation is to verify its results in various scenarios. We initiated our central Server and started to listen to requests from Clients. One after another, the nodes started sending connection requests, which the Server accepted, thus creating a multi-client scenario where routing and forwarding took place automatically using the BATMAN-ADV protocol. Since the Raspberry Pies are Linux-based, we confirmed the communication through a built-in network diagnostic tool called “traceroute”. This command shows the entire path taken by a packet from one end to another. It utilizes an IP protocol’s Time To Live (TTL) field and attempts to elicit an ICMP TIME_EXCEEDED response from each node along the path to the destination.

#### 4.2.1. Scenario 1: Client–Server Direct Communication

Firstly, the user only gets connected to the Control Room without the need for any intermediate entity, as both are within the range of each other. This can be verified through the following command on the Linux terminal:

“$ sudo batctl originators”

**Figure 6:**
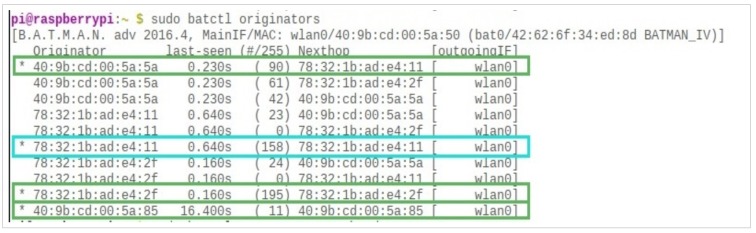
List of all available connections through the wlan0 interface.

This shows the list of all available routes, highlighted in purple in Figure 6. Since the BATMAN-ADV protocol works with Medium Access Control (MAC) addresses rather than IP addresses, all the connections are shown in terms of their MAC IDs. One can see that there is a direct path to the server (with a MAC address of 78 : 32 : 1*b* : *ad* : *e*4 : 11 shown in red. The path quality is 158 (out of 255), indicating a rather strong connection, and therefore no intermediate hop is required for the packet to reach its destination.

**Figure 7:**
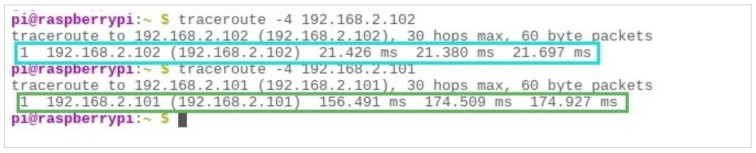
One-to-One TCP communication between the user and Control Room.

As can be seen in Figure 7, when the client (192.168.2.103) checks its path to the server (192.168.2.102), it receives a response in around 21 milliseconds, highlighted in red. Although there is an alternate path in the network, where there is a node with an IPv4 address of (192.168.2.101), as shown in purple box. However, it has a longer route taking at least 150 milliseconds (high link cost) to traverse. Therefore, the client would pick the shorter route (low link cost) to send its message.

#### 4.2.2. Scenario 2: Forwarding through One Intermediate Hop

**Figure 8:**
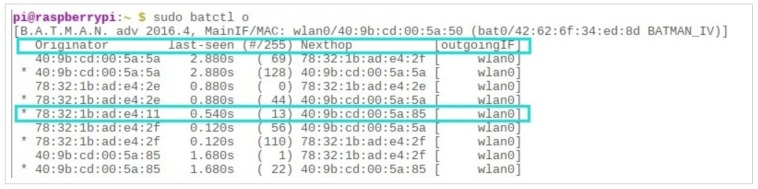
One hop through the intermediate (40 : 9*b* : *cd* : 00 : 5*a* : 85) node.

As the user is mobile, there will be a time when it gets away from the server’s range and the established path will not be available anymore. The user now needs other nodes to forward its packets to the server. The user, however, is still quite close to the server—therefore, just one hop will be sufficient for the communication to take place, as presented in Figure 8.

**Figure 9:**
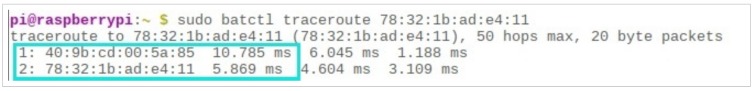
Total travel time for the packet is the sum of both the hops.

When we trace the route of a packet toward the server with a MAC ID 78 : 32 : 1*b* : *ad* : *e*4 : 11, we see that it takes one hop to an intermediate node (with a MAC ID of (40 : 9*b* : *cd* : 00 : 5*a* : 85) in around 10.78 milliseconds, and then travels for another 5.87 milliseconds to reach the server. The total time for the packet to complete its journey is then roughly 16.65 milliseconds, as depicted in Figure 9.

#### 4.2.3. Scenario 3: Forwarding through Multiple Intermediate Hops

**Figure 10:**
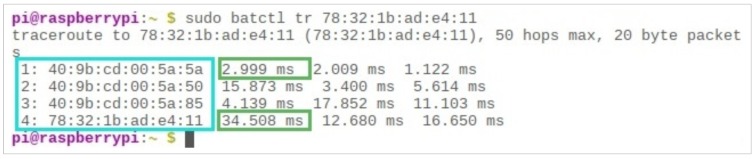
Four-hop scenario, three intermediate nodes.

Now if we increase the distance between the two endpoints—that is, the server and user—there will be more hops in between, and the travel time for a packet will increase. Figure 10 shows such a scenario where the packet takes four hops in 57.5 milliseconds to reach the destination. It can also be noticed that some of the nodes are at a greater distance from each other, thus requiring a longer time for packets to travel, for instance, 34.5 milliseconds, while others might be closer and take less time for a packet to travel, such as only 3 milliseconds, as shown in Figure 10 with the purple boxes.

**Figure 11:**
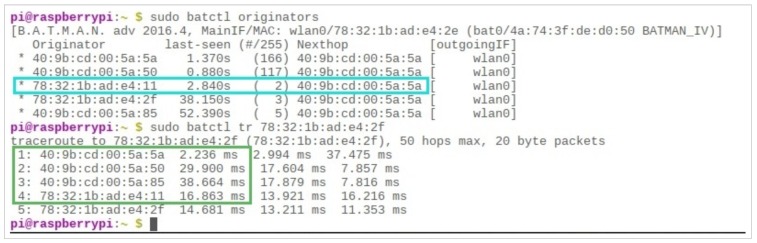
One-hop link quality is merely 2 out of 255, max.

BATMAN-ADV is an intelligent protocol that has link quality awareness. When confronted with the situation of more than one route to the destination, it will automatically pick the one with the best link strength and least travel time. One such scenario is presented in Figure 11. Although there is a one-hop route towards the server (through a node with MAC Address (40 : 9*b* : *cd* : 00 : 5*a* : 5*a*), the link quality of the said path is almost non-existent. Hence, the BATMAN-ADV. protocol will take four hops to reach the destination in order to make sure that the message is properly delivered in its entirety.

**Figure 12:**
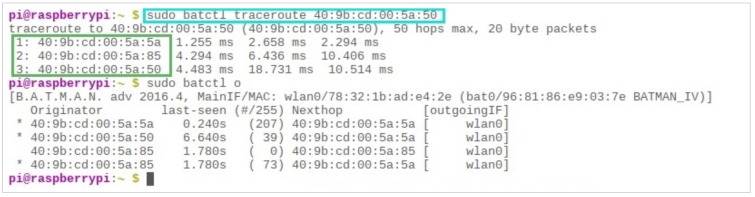
Trace route at Server for a node with MAC Address 40 : 9*b* : *cd* : 00 : 5*a* : 50.

We tested the delivery of packets from a certain client to the server, through multiple hops. However, one should be aware that the TCP communication is bi-directional—not only can multiple users send messages to the server, but the server can also reply to any user via a message in the opposite direction of propagation, as shown in Figure 12, where a packet from the server takes three hops to reach the end-user.

**Figure 13:**
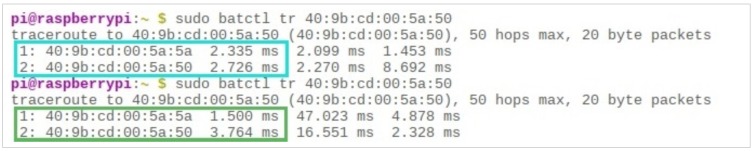
The dynamic nature of mobile users, indicated through the “batctl” traceroute.

It should also be noted that, since the entire communication chain is dynamic, the travel time for packets from any particular node to reach any other is constantly changing. As can be seen in Figure 13, the packet sent to a node (40 : 9*b* : *cd* : 00 : 5*a* : 50) took 2.33 and 2.72 milliseconds for its two consecutive hops, respectively. When another packet was sent from the same node to the same destination, it took less time for the first hop (1.5 milliseconds) and more time for the second hop (3.76 milliseconds), indicating the mobility of its users.

**Figure 14:**
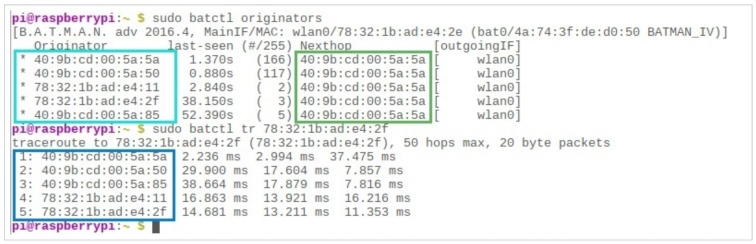
The same next-hop for all five available users.

One limitation of the “$sudo batctl originators” command, as shown in Figure 14, is that it only shows the next hop toward any direction of propagation—although there are five different users in the vicinity, the next hop for any one of those five routes is the same. However, in order to view the end-to-end path, the “$sudo batctl tr” command can be used with the MAC address of the destination.

**Figure 15:**
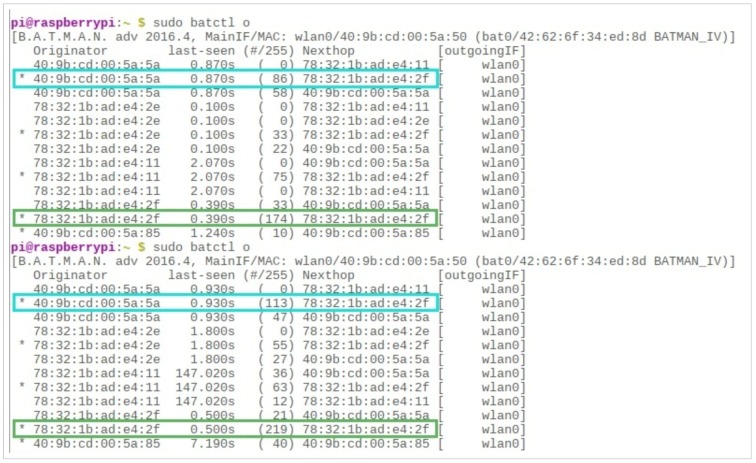
Dynamism of BATMAN-ADV Protocol.

Lastly, it is worth noting that BATMAN-ADV is a highly dynamic protocol which continuously monitors the environment and keeps itself updated. Therefore, communication is realistic, and ensures the best possible quality at any given time. As shown in Figure 15, each time we ran the “$sudo batctl o” command, it assessed the quality of each available path in the network and updated the relevant values. For example, a path which had a low link-quality of 86 in the first run had a link strength of 113 the next time we ran the same command (highlighted in red). Similarly, another path with a link quality of 174 had an even greater link strength of 219 the second time round (highlighted in purple).

### 4.3. Emergency Response System: Real-Time Statistics

**Figure 16:**
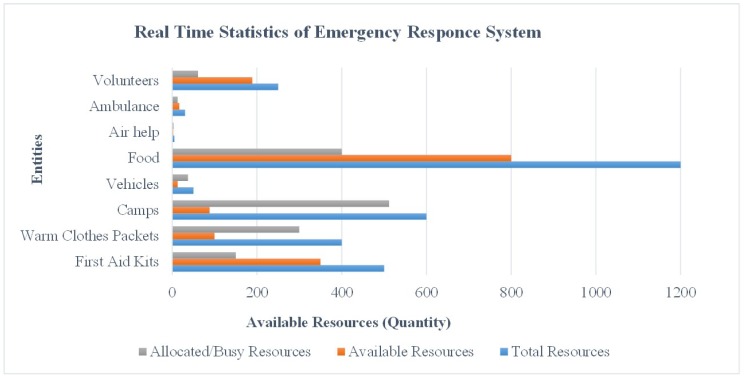
Emergency response system—the status of the inventory after receiving 200 queries from the disaster area.

**Figure 17:**
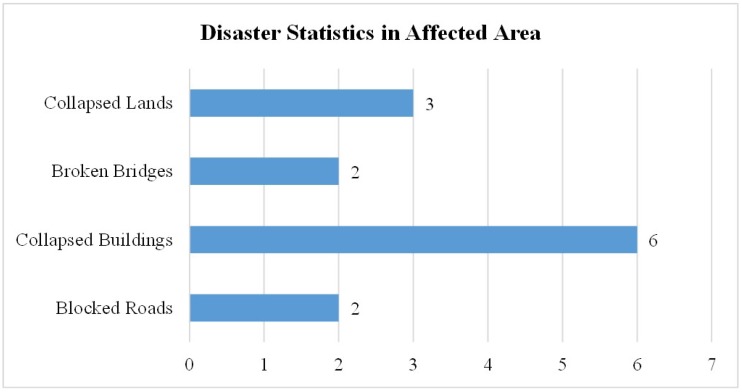
Emergency response system—the level of disaster in a particular area in terms of destruction.

In each aforementioned scenario, queries were generated in order to check the statistics displayed on the server side at the control room. Figure 5 presents the user interface at the server side that shows the disaster statistics, activity logs, and resource entities available in the inventory, to yield the facilitation to the affected people. After generating 200 queries, the system presented real-time statistics against the available resources in the inventory and the disaster statistics for the investigated scenario. It is assumed that the disaster occurred due to an earthquake, which has destroyed or demolished the already deployed communication infrastructure like LTE, or 3G/4G. Additionally, the proposed system was tested with the presumption that the volunteers are dispersed in the affected area. Since the system is designed to support the rescue operation for post-disasters, it is thus assumed that the rescue operation is well-prepared with basic entities to handle critical life threats. Hence, it is equally important to have information of available and allocated resources in the inventory. Based on this assumption, the proposed system gives real-time statistics about the available and allocated/busy resources on the user-interface at the server side in the control room. Figure 16 depicts the status of the inventory that is maintained to cope with the challenges of post-disaster management. This chart shows the quantity of resources on an *x*-axis and available entities on the *y*-axis. It provides the information of total resources indicated with the blue bar, available resources with the red bar, and allocated/busy resources with the grey bar. This may vary in different scenarios depending on the need and the level of disaster in a particular area. Figure 17 presents the incident reported via emergency messages to the control room. These incidents include collapsed lands, broken bridges, collapsed buildings, and blocked roads. The increased number of reported events indicates the level of disaster.

The results in the following section show that the system performed all the above-mentioned tasks. The performance of the system was analyzed in terms of response time, successful data transmission, and delay. However, the implemented system was tested in a lab under the constraints of a limited availability of hardware and testing area.

### 4.4. Performance Analysis

**Figure 18:**
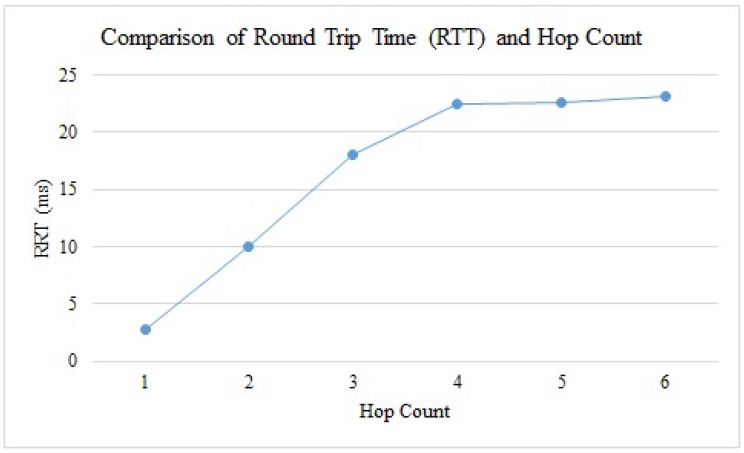
Analysis: Round Trip Time (RTT) vs Hop Count.

It is always a good idea to compare different scenarios and to produce conclusive results, in order to better understand the processes of any system. This analysis not only helps in decision-making, but also helps to find solutions for any current or future issue. We plotted the average values of the Round Trip Time (RTT) taken by sent packets in various executions of the system, versus the number of hops they traversed. Obviously, the greater the number of hops, the more time it takes for the data to reach its destination. We witnessed that by executing the same operation by changing the positions of the users (robot cars, in our case) every time, the values for the RTT changed accordingly, as depicted in Figure 18. This change is mainly due to the mobility of the cars, but is due also in part to the disturbances in the wireless environment (e.g., scattering effects, interference with other signals).

**Figure 19:**
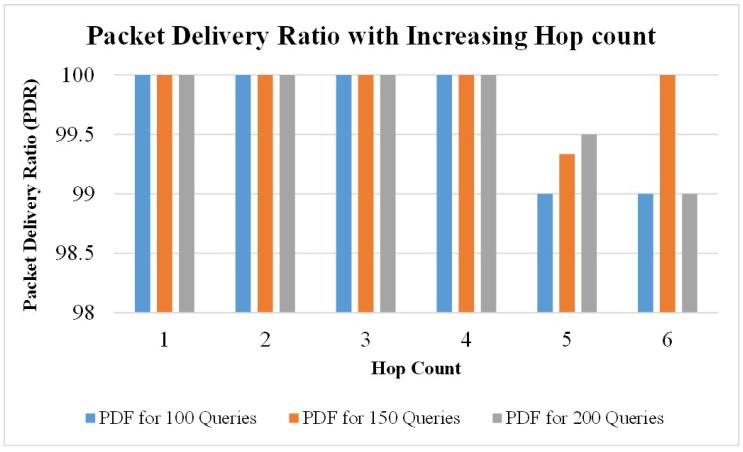
The trend of packet delivery ratio (%) with increasing hop count.

Additionally, we performed three different tests by executing the proposed applications on the TestBed. 100, 150, and 200 queries were generated for each hop-count value (i.e., 1, 2, 3, 4, 5, 6 in the first, second, and third test, respectively). In each test, the Packet Delivery Ratio (PDR) was observed by increasing the hop-count value, as shown in Figure 19. It was observed that PDR was 100% with a hop-count value from 1 to 4, which shows that all the queries that were sent to the intended destination were successfully received. However, with an increase in the number of hop-counts greater than 4, the PDR decreased to some extent, showing that there was a loss in the received queries which were sent by the sender. This happened possibly because of interference due to the increase in the number of nodes in any wireless communication.

**Figure 20:**
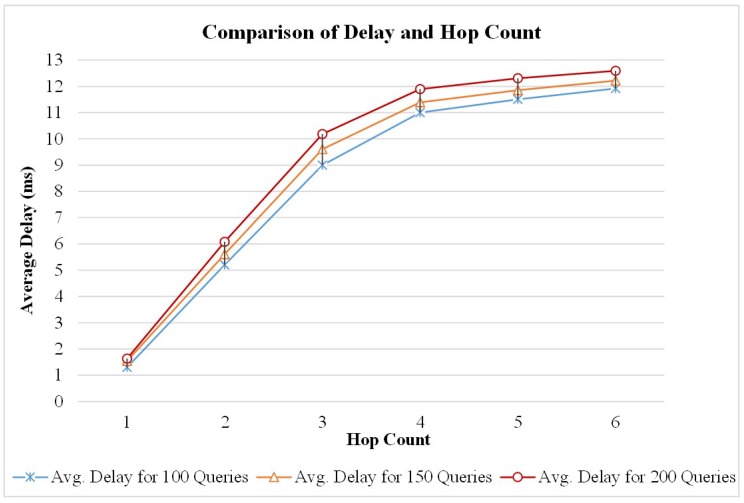
Comparison of Delay with Increasing Hop Count.

It is to be noted that safety and emergency applications require a 20–100 ms delay. Though the proposed application is designed to meet the communication requirement in an emergency situation, the nature of the application can tolerate little delay. During the testing phase, the average delay was evaluated for the queries. Figure 20 presents the average delay comparison with the number of hops. The TestBed encompasses a limited number of vehicular nodes, and the observed delay is below the minimum requirement of the safety application.

This concludes our work toward presenting a real-world prototype, which comes with its own set of advantages and disadvantages. This work can be considered to realize even better systems that can be commercially used in rescue-and-relief efforts in disaster-management areas.

## 5. Conclusions and Future Work

The prime goals of the proposed work were attained with the complete design of an emergency response system and with the successful implementation of a TestBed, which carried out the intended communication using VANET. Our study provided ways in which the scientific society could achieve the required goals and objectives of an emergency response system applicable in a vehicular environment, and presented real-time statistics of an emergency response system along with the disaster statistics of an affected area. Furthermore, it provided a way to measure the performance of a network in terms of round-trip-time with reference to the number of hops along the route from the source to destination. Moreover, the research work may also be used to receive knowledge about reliability, and delay factors based on the hop-count value along the route within the network. The proposed prototype application was examined through experimental validation by fetching the user message from the GPIO pins of the Raspberry Pi through serial communication, and the geographical coordinates of the user from the GPS sensor. The communication was performed using the Transmission Control Protocol, mainly because of its connection-oriented and reliable nature. Out of all the commercially available ad hoc routing protocols, BATMAN-ADV was selected because of its sophisticated abstraction. It hides all lower-level complexities and provides an easy-to-use virtual interface between Layers 2 and 3 (Network and Data Link, respectively) of the TCP/IP model. This study, along with the implemented TestBed, provides an effective way to exchange crucial information amongst volunteers and staff working in dire situations, such as that of a post-disaster rescue-and-relief operation. Since fast and reliable communication plays a key role in such scenarios, this work offers a viable solution that can be implemented by the concerned authorities at times of catastrophic disaster.

## Figures and Tables

**Table 1:** Alert messages, their types, and Transferred Message Codes.

No.	Alert	Type	Message Code
1	First Aid	Emergency Help Call	1
2	Ambulance	Emergency Help Call	2
3	Air-help	Emergency Help Call	3
4	Food/Water	Help Call	4
5	Vehicles	Help Call	5
6	Shelter/Camp	Help Call	6
7	Volunteers	Help Call	7
8	Warm-clothes	Help Call	8
9	Collapsed Land	Informative	9
10	Broken Bridge	Informative	A
11	Blocked Road	Informative	B
12	Collapsed Buildings	Informative	C
13	All OK/Clear	Informative	D

**Table 2:** Summary of actions taken by the Server at the Control Room.

Code	Action
1	Sends a message to the First-Aid team nearest to the location
2	Sends a message to the Hospital service
3	Sends a message to the Air-Base Control room
4	Sends a message to the Food & Accessories management head, along with location information
5	Sends a message to the Transport service head, along with location information
6	Sends a message to the Camping & Shelter management head, along with location information
7	Sends a message to the Volunteers management center asking for volunteers at the specified location
8	Sends a message to the Food & Accessories management head, along with location information
9	Stores the information in database and displays it on the User Interface
A	Stores the information in database and displays it on the User Interface
B	Stores the information in database and displays it on the User Interface
C	Stores the information in database and displays it on the User Interface
D	No action is performed as this only acknowledges the end of the message

## Abbreviations

The following abbreviations are used in this manuscript:

AP	Access Point
BATMAN	Better Approach To Mobile Ad hoc Networking
CTOS	Commercial off-the-shelf
CPU	Central Processing Unit
DSRC	Direct Short Range Communication
EPIC	Explicitly Parallel Instruction Computing
GPS	Global Positioning System
GUI	Graphical User Interface
GoPiGo	General Purpose Input Output
ID	Identity
I2I	Infrastructure-Infrastructure communication
IBSS	Independent Basic Service Set
ILS	Indoor Localization System
IECS	Integrated Emergency Communication System
IESS	Integrated Emergency Service System
ISE	Integrated Synthesis Environment
JSON	Java Script Object Notation
LOS	line-of-sight
MAC	Medium Access Control
MANET	Mobile Ad hoc Network
MAPs	Mesh Access Points
MCs	Mesh Clients
OSI	Open Systems Interconnection
OBU	On-Board Unit
OS	Operating System
OSI	Open Systems Interconnection
P2Pnet	peer-to-peer communication network
FPGA	Programmable Gate Array
PDAs	Personal Digital Assistants
RSU	Road Side Unit
RAM	Random Access Memory
RNs	Relay Nodes
RFID	Radio-frequency identification
RISED	Rescue Information System for Earthquake Disasters
SUMO	Simulation of Urban MObility
TCP	Transmission Control Protocol
TCP/IP	Transmission Control Protocol/Internet Protocol
TTL	Time To Live
VANETs	Vehicular Adhoc Networks
V2I	Vehicle-to-Interface
V2V	Vehicle-to-Vehicle
WAVE	Wireless Access in Vehicular Networks
WMN	Wireless Mesh Networks
WSN	Wireless Sensor Network
UNISDR	United Nations Office for Disaster Risk Reduction
